# Case-control study of failed extubation

**DOI:** 10.1186/cc10735

**Published:** 2012-03-20

**Authors:** J Krinsley, P Reddy, A Iqbal

**Affiliations:** 1 Stamford Hospital, Stamford, CT, USA

## Introduction

Failed extubation (FE), defined as reintubation within 48 hours of planned extubation (PE), is common. The literature suggests that FE complicates 10 to 20% of PE. The consequences of FE have not been well described, nor have its risk factors.

## Methods

We performed a retrospective study of prospectively collected data involving 2,012 consecutive patients undergoing mechanical ventilation (MV) in a 16-bed university-affiliated hospital between 1 October 2005 and 31 August 2011. Eighty-five patients with FE were matched 1:3 with successfully extubated patients (SE) using diagnostic category, age, Acute Physiology Score (APS) and duration of ventilation (DOV) before PE as matching criteria.

## Results

Patients undergoing MV included 1,209 (60.1%) with SE; 224 (11.1%) died during ventilation (without prior FE); 206 (10.2%) were extubated to withdraw support; 180 (8.9%) were transferred from the ICU while ventilated; 81 (4.0%) were liberated from MV after tracheostomy; 85 (6.6%) failed PE. APS scores were higher (53 (42 to 69) vs. 43 (32 to 60), *P *< 0.0001) and DOV before PE longer (1.8 (0.8 to 4.4) vs. 0.9 (0.4 to 2.6), *P *= 0.0001) in FE than in SE. There was 100% concordance of diagnostic category and no statistically significant differences between the groups in regards to age, APS and DOV before PE. Table 1 illustrates the results of the case-control analysis. In addition, FE had more days in the hospital after ICU discharge than did SE: 11 (4 to 24) versus 5 (2 to 9), *P *< 0.0001.

**Table 1:** Case-control analysis of failed extubation: key outcomes

	FE	SE	*P *value
ICU LOS	11.8 (7.7 to 17.5)	3.8 (2.1 to 7.5)	<0.0001
VAP (%)	7.1	0.8	0.0043
Mortality (%)	23.5	10.2	0.0052

## Conclusion

FE is associated with increased ICU and hospital LOS, increased risk of VAP and increased mortality. Efforts to prospectively identify patients at risk for FE may reduce its incidence and improve outcomes.

